# Appropriateness and diagnostic yield of cardiac magnetic resonance imaging from a tertiary referral center in the Middle East

**DOI:** 10.1186/1532-429X-17-S1-P303

**Published:** 2015-02-03

**Authors:** Wael AlJaroudi, Hussain Ism'eel, Fadi El-Merhi, Tony E Assaad, Mukbil Hourani

**Affiliations:** 1 AUBMC, Beirut, Lebanon

## Background

Cardiac magnetic resonance imaging (CMR) is a novel non-invasive modality with many potential indications, and was recently introduced in Lebanon. We sought to assess the appropriateness and diagnostic yield of CMR studies performed at a tertiary referral center from the Middle East since the inception of the program.

## Methods

All patients who underwent CMR studies between January 1^st^ 2013 and June 18^th^ 2014 were enrolled in this study. CMR reports were retrospectively reviewed. The study indication, clinical history, and findings were extracted and analyzed by a level III trained cardiologist. The appropriateness of the study was judged according to the 2010 updated Asian Society of Cardiac Imaging guidelines.

## Results

**Figure 1:**
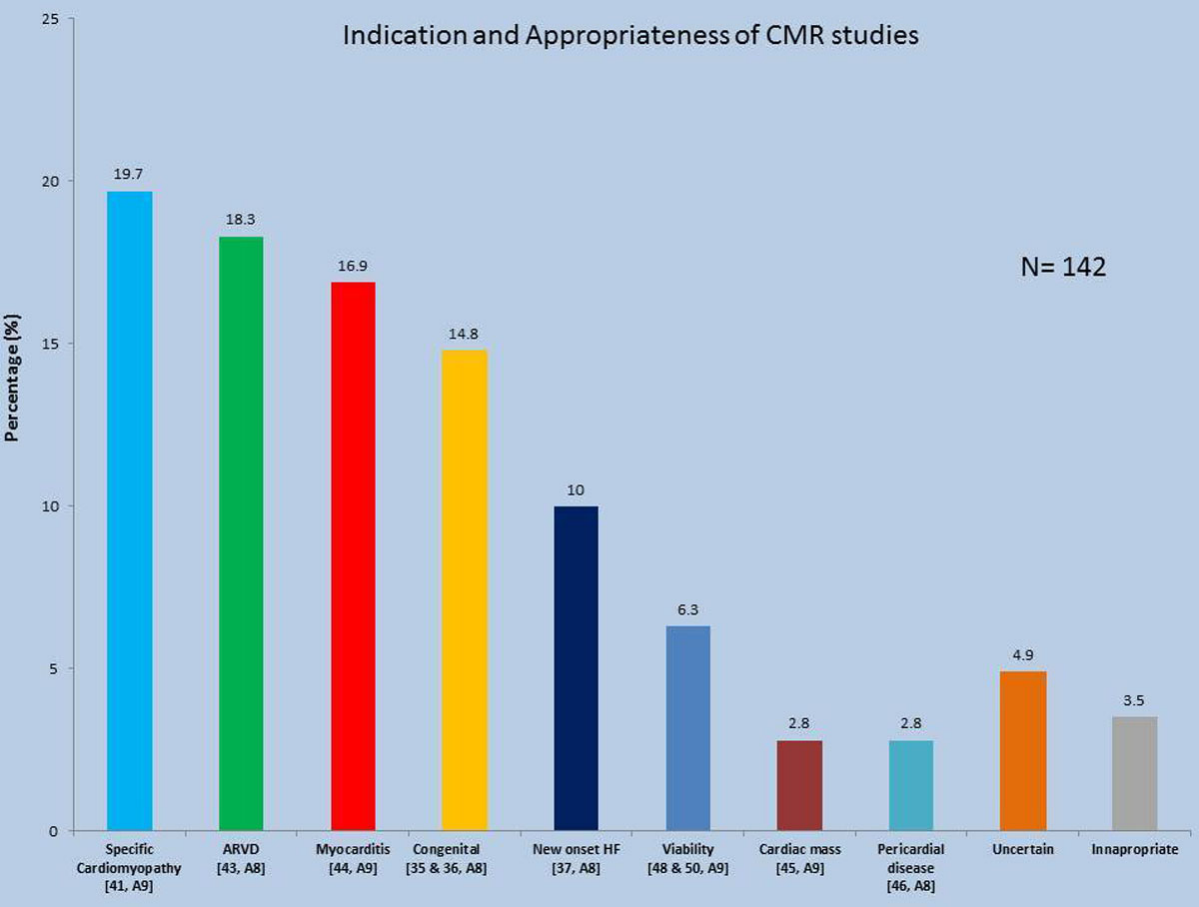


There were a total of 142 patients (mean age [SD] 42.1 [18] years, 24.6% female) that underwent CMR study. Two-third of studies were performed on an outpatient basis, and outside referrals constituted 16.2% of the entire cohort. The cardiologists referred 122 cases (86%) with main contribution from electrophysiology and imaging specialists. Of the 142 cases, 12 (8.4%) were not indicated and added little value (Table 1). Of the remaining 130 appropriate studies (appropriateness level A8-A9) (Figure 1), one-third had an incorrect diagnosis prior to CMR, and 8% had relevant findings that were missed on other studies but captured by CMR. Furthermore, CMR confirmed the diagnosis in 28% of the cases, provided relevant information on scar burden, shunt quantification, and ruled out infiltrative disease in the remaining patients. Also, CMR demonstrated the presence of scar in 45 of patients, among whom 20 (44%) had significant burden (>5% of left ventricular myocardium). Finally, 9% of patients had a relevant extra-cardiac finding that needed further investigation.

**Table 1:** Details of non-appropriate indications for CMR studies

Original indication	Appropriateness	Details
44-Myocarditis	Uncertain	Patient had prior CMR showing myocarditis (LGE 15% LV) and mildly reduced EF. Follow-up echocardiogram showed normal EF. Follow-up CMR was ordered to assess residual LGE for risk stratification

44-Myocarditis	Uncertain	Patient had prior CMR showing myocarditis (LGE 10% LV) and normal EF. Follow-up CMR was ordered to assess residual LGE for risk stratification

43-ARVD	Uncertain	Positive family history of SCD. Normal echocardiogram. Concern for ARVD. No syncope or VT.

43-ARVD	Uncertain	Patient with VT. Normal echocardiogram. CMR ordered to rule out scar or focus for arrhythmia

41-Specific cardiomyopathy	Uncertain	Palpitation and presyncope. Normal echocardiogram. Rule out scar or infiltrative disease

43-ARVD	Uncertain	Patient had prior CMR showing minor ARVD criteria with frequent PVCs and low EF. CMR ordered post ablation to assess for scar at the site of ablation and improvement in EF.

38-LVEF in heart failure	Uncertain	Patient had an echocardiogram- did not add much information.

45-Mass	Inappropriate	Echocardiogram clearly showed prominent Chiari network and not right atrial mass

43-ARVD	Inappropriate	Dizziness-concern for ARVD. Normal echocardiogram. No syncope or VT

43-ARVD	Inappropriate	Brother died while swimming. R/o ARVD. Normal echocardiogram. No VT or syncope

43-ARVD	Inappropriate	Palpitation with normal echocardiogram. Rule out scar or fibrosis

46-Pericardial disease	Inappropriate	First episode of pericarditis. Negative cardiac enzymes. CMR ordered to assess for pericardial and myocardial LGE

ARVD (arrhythmogenic ventricular dysplasia); CMR (cardiac magnetic resonance imaging); EF (ejection fraction); LGE (late Gadolinium enhancement); LV (left ventricle); SCD (sudden cardiac death); VT (ventricular tachycardia)

## Conclusions

Despite the recent launch of the CMR program at our institution, the majority of studies were appropriately indicated, provided relevant data and were clinically useful. Inappropriate or uncertain studies did not provide relevant data, and should be further minimized to avoid unnecessary costs and downstream testing. Large prospective CMR database with clinical follow-up is needed to provide more insight about cardiovascular disease and outcomes in our population.

## Funding

None.

